# Federated Learning for Privacy-Aware Human Mobility Modeling

**DOI:** 10.3389/frai.2022.867046

**Published:** 2022-06-28

**Authors:** Castro Elizondo Jose Ezequiel, Martin Gjoreski, Marc Langheinrich

**Affiliations:** Faculty of Informatics, Università Della Svizzera Italiana, Lugano, Switzerland

**Keywords:** federated learning, mobility modeling, privacy, deep learning, location data

## Abstract

Human mobility modeling is a complex yet essential subject of study related to modeling important spatiotemporal events, including traffic, disease spreading, and customized directions and recommendations. While spatiotemporal data can be collected easily *via* smartphones, current state-of-the-art deep learning methods require vast amounts of such privacy-sensitive data to generate useful models. This work investigates the creation of spatiotemporal models using a Federated Learning (FL) approach—a machine learning technique that avoids sharing personal data with centralized servers. More specifically, we examine three centralized models for next-place prediction: a simple Gated Recurrent Unit (GRU) model, as well as two state-of-the-art centralized approaches, Flashback and DeepMove. Flashback is a Recurrent Neural Network (RNN) that utilizes historical hidden states with similar context as the current spatiotemporal context to improve performance. DeepMove is an attentional RNN that aims to capture human mobility's regularity while coping with data sparsity. We then implemented models based on FL for the two best-performing centralized models. We compared the performance of all models using two large public datasets: Foursquare (9,450 million check-ins, February 2009 to October 2010) and Gowalla (3,300 million check-ins, April 2012 to January 2014). We first replicated the performance of both Flashback and DeepMove, as reported in the original studies, and compared them to the simple GRU model. Flashback and GRU proved to be the best performing centralized models, so we further explored both in FL scenarios, including several parameters such as the number of clients, rounds, and epochs. Our results indicated that the training process of the federated models was less stable, i.e., the FL versions of both Flashback and GRU tended to have higher variability in the loss curves. The higher variability led to a slower convergence and thus a poorer performance when compared to the corresponding centralized models. Model performance was also highly influenced by the number of federated clients and the sparsity of the evaluation dataset. We additionally provide insights into the technical challenges of applying FL to state-of-the-art deep learning methods for human mobility.

## Introduction

The study of human mobility patterns is a complex yet important subject. Human mobility can reveal many human behavioral characteristics and predict important spatiotemporal events, such as traffic congestions, the spreading of diseases, or public transportation demands. However, human mobility modeling is far from being an easy task. Feng et al. (2018) classifies the unique considerations for mobility modeling systems into three different groups:

- *Complex time-dependent sequential transitions*. Given the many unknown factors that influence a mobility pattern (e.g., available means of transportation, job priorities, family-imposed restrictions), human trajectories are often approximated using random-walk or diffusion models (Gonzalez et al., 2008). Holidays and weekends see even more irregular mobility traces as the usual transition between workplace and home disappears.
- *Multi-level periodicity of human mobility*. Mobility periodicity is often complex and multi-level, merging daily, weekly and yearly activities with other personal periodic activities. While typical mobility models can describe transitional regularities well, more complex multi-level periodicity is still hard to capture.
- *Heterogeneity and sparsity in trajectory data*. Much of today's spatiotemporal information only gets recorded when the user decides to share it through a particular application (e.g., a social media platform like Foursquare, Twitter, or Facebook). This low sampling rate makes it harder to train individual mobility models. At the same time, the heterogeneous mobility habits of different users make it hard to train general models.

Because of the constantly increasing amount of available data, the improvements in computational capacity, and the predictive power that machine learning (ML) models provide, ML-based approaches have become an essential tool for human mobility modeling. Traditional ML approaches such as probability models, pattern-based models, autoregressive models, and more advanced deep learning models have been successfully applied to problems like traffic prediction, personalized Next-Point-of-Interest (POI) prediction, and crowd flow prediction. However, the centralized nature of these ML approaches quickly raises privacy concerns, as user data from multiple devices is shared with a server to train a general (joint) model. Alternatively, one can build a personal model directly on a user's device, based only on this user's data, without needing centralized servers. Personal models improve privacy, yet this typically comes at the expense of predictive performance (Feng et al., 2020).

## Contributions

We base our work on two centralized state-of-the-art end-to-end deep learning models for next-place prediction:

- Flashback (Yang et al., 2020) is a Recurrent Neural Network (RNN) model that uses a spatiotemporal weight to look for similar trajectories for the next POI prediction problem. Flashback explicitly uses spatiotemporal context to search past hidden states with high predictive power (Yang et al., 2020). In this way, the model does “flashbacks” on the RNN's hidden states to determine the relevance of the current input according to similar historical contexts. Flashback outperformed related RNNs by raising the obtained accuracy from 15.9 to 27.6% in the next-place prediction task.
- DeepMove (Feng et al., 2018) is an attentional RNN specialized for sparse data that spans extended periods (e.g., close to a year in the Flashback dataset). DeepMove uses an attention mechanism to extract mobility patterns from historical trajectories, while Gated Recurrent Units (GRUs) handle current trajectories (Luca et al., 2020). The authors report that DeepMove outperforms related state-of-the-art models by more than 10% in the next-place prediction task.

We first replicate the experimental results achieved in the original Flashback and DeepMove studies. We then explore the feasibility of privacy-aware next-place prediction by implementing predictive models using Federated Learning (FL). This work presents the following six contributions:

1. Implementation of a baseline model for human mobility modeling: GRU-Spatial. This model represents a baseline RNN architecture against which we compare the advanced models.
2. A centralized implementation of two state-of-the-art deep learning architectures, Flashback and DeepMove
3. A replication of the original Flashback and DeepMove results and their comparison to GRU-Spatial in a centralized learning environment
4. Novel FL implementations of the two best performing deep learning models: GRU-Spatial and Flashback
5. A comparison of GRU-Spatial and Flashback in a centralized and federated context using two large public datasets, Foursquare and Gowalla
6. First insights into open challenges for applying FL on deep learning approaches for human mobility modeling.

### Paper Structure

The rest of this paper is structured as follows: the following section presents related work on human mobility modeling, including Markov models, feature-based ML models, and end-to-end deep learning models. We then describe the two experimental datasets used in this study, followed by a technical description of the methods used to develop the predictive models (GRU architecture, DeepMove, Flashback, and FL). Next, we present the experimental setup and the experimental results. We close with a discussion section and conclusions that summarize our key insights.

## Related Work

### Markov Models for Mobility Modeling

Mobility Markov models compute a state transition matrix that models POIs as the states and every transition among them. These transitions are collected from a user during a period of time and are the input to the model. After forming the state transition matrix, the predicted next POI can be identified as the most likely state according to the calculated transition probabilities (Kulkarni et al., 2016). Ashbrook and Starner (2002, 2003) used a first-order Markov model to predict user-specific future movements. Song et al. (2004) showed that a second-order Markov model might be better than first-order predictors. Imai et al. (2018) proposed an improvement to the Markov-based approaches, where the set of possible places to be visited narrows down as the trip progresses.

### Feature-Based Models for Mobility Modeling

Baumann et al. (2013) analyzed a variety of spatial features (e.g., current location and previous location) and temporal features (e.g., day of the week and weekday/weekend) as possible next-place predictors using data of 37 users collected over 1.5 years. Jindal et al. (2017) used a multi-layer perceptron to estimate the distance and the duration of taxi trips. Bhyri et al. (2015) proposed a multi-level approach by predicting the semantics of a place and then the specific place to be visited. The feature sets used in these studies include current location, last call, the hour of the day, day of the week, and used smartphone applications. Similarly, Prabhala and La Porta (2015) used start minute, end minute, normalized start time, and other related features as input to a Support Vector Machine classifier. Etter et al. (2012) compared a variety of methods, including a majority classifier (35% accuracy), first-order Markov model (44% accuracy), deep belief network (60.7% accuracy), neural network (60.83% accuracy), and gradient boosting trees (57.63% accuracy).

### End-to-End Deep Learning Models for Mobility Modeling

The most recent and advanced next-place predictors are based on end-to-end deep learning methods. ST-RNN (Spatial-Temporal Recurrent Neural Networks), DeepMove (Feng et al., 2018), RNN+SAtl (Zeng et al., 2019), and Flashback (Yang et al., 2020) are all based on RNNs or their variations (e.g., LSTMs or GRUs). Our work is based on DeepMove and Flashback—we will explain these architectures in more detail in the Methods section below. Nevertheless, all these methods use personal data to train centralized models, which carries significant privacy implications.

### Federated Learning

FL allows devices to learn a shared model collaboratively while keeping all the training data on-device (Google, 2017). An example application that benefits from FL is Gboard—Google's onscreen keyboard application on Android phones. Gboard uses FL to improve its next-word suggestion model by merging local prediction with a global model shared with other participating Android phones using a differential privacy method. Each involved Android device contains a light-weight version of TensorFlow (Abadi et al., 2016) and uses an intelligent scheduler to make sure that the phone only trains a model when idle, plugged in, and is connected to WiFi (to avoid cellular data charges).

FL has been used in a variety of domains. Tian et al. (2022) used FL in natural language processing for training a federated version of a large-scale language model (BERT). Their approach enables pre-training large-scale models without having big data at one centralized server. Brisimi et al. (2018) applied FL on electronic health records to develop predictive models for heart-related hospitalizations. Ek et al. (2021) and Sozinov et al. (2018) used data from wearable sensors (e.g., smartphones and smartwatches) to develop activity recognition models. Dayan et al. (2021) used FL for predicting clinical outcomes in patients with COVID-19. They used data from 20 medical institutions to train an FL model that predicts the future oxygen requirements of symptomatic patients with COVID-19. The model used structured data from electronic health records and chest X-ray data. Similarly, Dou et al. (2021) applied FL on data from 132 patients from seven multinational medical centers from Hong Kong, Mainland China, and Germany to develop models for detecting COVID-19 lung abnormalities in Computerized Tomography (CT) scans. Rey et al. (2022) developed FL framework for malware detection in IoT devices. The framework enables the training and testing of both supervised and unsupervised models. Similarly, Liu et al. (2020) developed attention-based DL models trained *via* federated learning for anomaly detection in industrial IoT data. The authors proposed a gradient compression mechanism based on top-k selection to improve communication efficiency. Jiang et al. (2020) presented an overview of FL for smart cities, including insights on open issues such as energy consumption, adversarial attacks, and data distribution (unbalanced quantity, features, and labels).

The growing applicability of FL has resulted in several tools and frameworks becoming available. These include TensorFlow Federated (Google, 2017), Flower (Beutel et al., 2020), and Leaf (Caldas et al., 2018). The usage of FL in many domains shows that it is a promising technique for privacy-aware ML. Nevertheless, FL has many open challenges to be addressed, including non-iid data, distributed and unbalanced data, unreliable connectivity, communication overhead, heterogeneous hardware, poor device performance, poisoning attacks, server-side attacks, federated optimization algorithms, federated model selection, and federated debugging (Kairouz et al., 2021; Yu et al., 2022).

### Federated Learning for Mobility Modeling

Feng et al. (2020) proposed PMF, a “privacy-preserving mobility prediction framework” that uses FL to train general mobility models in a privacy-aware manner. In PMF, every participating device trains locally a representation of the global (centralized) model by using only the locally available dataset at each device. The produced weights are then individually encrypted and uploaded to the server for aggregation. The system then generates a new model representation and downloads it to the participating devices before the process repeats until convergence. The PMF methodology aims to avoid collecting private data by sharing only the resulting gradients of each personal model, which are harder to decode in case of an attack.

While PMF demonstrates the feasibility of using FL for human mobility modeling, it is unclear whether specialized federated architectures are needed or if any deep learning architecture can be used. For that reason, we investigate whether we can easily integrate existing centralized state-of-the-art deep learning architectures with FL for privacy-aware human mobility modeling.

As PMF was not publicly available, we could not perform a direct experimental comparison with this architecture. Nevertheless, the result reported on the Foursquare dataset in the PMF study (Top-1 accuracy of 21%) is similar to the Foursquare results in our work (Top-1 accuracies of 23–26% achieved by the centralized models). Because of the variations in the pre-processing steps and train/test dataset splits, these results are not directly comparable between the two studies (we followed the experimental setup from Flashback). Nevertheless, they converge on a similar best performance reported in the literature.

## Datasets and Methods

### Datasets

Location-Based Social Networks (LBSNs) have become an important tool to gather valuable data for understanding social interactions and trends in social behaviors. In a typical LBSN, users voluntarily “check-in” at specific places (e.g., restaurants), thus contributing a spatiotemporal data point to their mobility record. Many pioneering LBSN platforms, such as Gowalla and Brightkite, are not active anymore, yet they live on in their publicly released datasets. Today's LBSN, such as Instagram or Facebook, closely guard their mobility data. A notable exception is Foursquare, which is still active and has released several datasets in the past. We use data from Gowalla and Foursquare in this work and will describe both datasets in the following sections. We obtained both datasets *via* the published Flashback data (Yang et al., 2020).

### Gowalla

Gowalla was an LBSN that started in 2007 and remained active until 2012. Gowalla users could voluntarily check-in at places (called “spots”) through a mobile application or a web page. The Gowalla dataset consists of more than six million check-ins of pseudonymized users from February 2009 to October 2010.

### Foursquare

Foursquare City Guide, also known just as Foursquare, is a local search-and-discovery mobile application developed by Foursquare Labs. This LBSN allows users to search and discover new places of interest according to their location, check-ins, and browser history. The Foursquare dataset contains nine and a half million check-ins from pseudonymized platform users between April 2012 and January 2014.

Table 1 shows a comparison between the Gowalla and Foursquare datasets. The Foursquare dataset has more check-ins—suggesting that Foursquare users were more active, while Gowalla has more users and more Points Of Interest (POIs). Given that Gowalla has more users but fewer check-ins (i.e., the Gowalla dataset is sparser) it should be harder to model.

**Table 1:** Gowalla and foursquare datasets comparison.

	**Gowalla**	**Foursquare**
#Users	52,979	46,065
#POIs	121,851	69,005
#Check-ins	3,300,986	9,450,342
Collection period	Feb/2009–Oct/2010	Apr/2012–Jan/2014
Avg time between check-ins	51.28 h	58.59 h

*Based on Yang et al. (2020)*.

### Data Pre-processing

Following the steps used in the Flashback study (Yang et al., 2020), we pre-processed both datasets in the same way and under the same criteria so that the experimental results achieved on the two datasets are comparable.

- **Data cleaning**. Users with incomplete data fields were removed. Occurrences of this were limited, since the nature of the data does not usually allow registration of incomplete data. That is, since in an LBSN, each data point is created by a specific user account at a particular place using geolocation methods, it is rare when the dataset does not contain a location tag together with both a timestamp and a user tag.
- **Data transformation**. The data of the original datasets required just a few simple transformations, for example, replacing both the user's and POI's IDs with integers. This is because POIs and users initially receive large numbers as IDs, so renaming them with identifications that started with 0 helps with both debugging and the classification output of the models. An important transformation to the original data was regarding the given timestamps. Temporal representation can be presented to the model using a simple linear mapping of values from 0 to 1 (normalization), but this will omit the cyclical nature of time. This simple mapping represents the same hour 0 and 24 as two different values, 0 and 1, respectively. This is a problem since the difference between the hours 23:59 and the hour 00:00 seems to be 23 h instead of only 1 min. To solve the problem and enrich the model with a notion of cyclical time, the mapping of the temporal data was slightly changed. The mapping of the time features was mapped using the following equation (Equation 1):

(1) $$\begin{array}{r} y_{1}\;=\;0.5\;*\text{sin}\left(\frac{2\pi h}{24}\right)\;+\;0.5 \end{array}$$

With the above equation, where *h* is the hour of the day, and *y*_1_ is the resulting mapping value of *h*, the cyclical nature of time is preserved. All models presented in this work depending directly on a temporal embedding, use this scheme to represent time. This is, of course, a simplification of the cyclical representation since the same value represents the hours 0:00, 12:00, and 24:00. This simplification is valid since, in the sparse datasets used for this work, a check-in is hardly present exactly at those hours. Hence, the model is expected to distinguish between check-ins performed in the morning (with values above 0.5) and check-ins done at noon (presenting values below 0.5). However, an additional modification has to be done to consider the hours 0:00 and 24:00 as the same but still different from 12:00 (noon). For this, an additional cosine function can be used to encode the time of the day. Using the following equation (Equation 2):


(2) $$\begin{array}{r} y_{2}\;=\;cos\left(\frac{2\pi h}{24}\right) \end{array}$$

Using sine and cosine encoding (*y*_1_, *y*_2_), the model can distinguish between noon and night easily.

- **Data reduction:** Users with <100 check-ins were removed from the datasets since a small number of check-ins can result in overfitting of one user's preferences, which can bias the final general model.
- **Data segmentation:** The pre-processed data was then segmented into sequences of 20 consecutive POIs. All models receive one sequence at a time and predict the next POIs visited by the user. The predictions were performed one at a time with no padding.

### Methods

This subsection presents the baseline model (GRU-Spatial) and two existing state-of-the-art models: Flashback by Yang et al. (2020) and DeepMove by Feng et al. (2018). In the last part, we present technical details for FL and the corresponding platform Flower (2021), which was used to develop the FL models.

The simplest architecture is the GRU-spatial architecture, which combines vanilla GRU architecture with two embedding layers. One embedding layer learns a dense representation of the user IDs, and the other one learns a dense representation of the POI IDs. The output of the two embedding layers is then fed to the GRU layers. The DeepMove architecture contains all the layers that the GRU-Spatial has, with an addition of an attention module. The attention module should enable the model to select relevant historical trajectories from the training data based on a presented input. The Flashback architecture is also based on RNNs and uses user embeddings. Unlike the other two architectures, Flashback uses specially designed weights that enable the model to measure the predictive power of all hidden states within the RNN network. The weights also allow the model to consider the temporal and spatial distances between the historical trajectories and the presented input. All three architectures feed the output of the recurrent layers (RNN or GRU) to a fully connected layer which then outputs probability estimations for next-place candidates that a specific user may visit.

### Baseline Architecture (GRU-Spatial)

A Recurrent Neural Network (RNN) is an artificial neural network specialized for sequential or time-series data. One of the most recognizable features of RNNs is their hidden state, which can be described as an output of state *i* to be converted into a part of the input for state *i* + 1. Because of this characteristic, the normal RNN considers past events alongside current ongoing inputs. This consideration of past events is then associated with “the memory” of the structure since the historical inputs act as a memory that can remember past events. RNNs suffer from two main problems: “exploding gradients” and “vanishing gradients”. Vanishing gradients occurs when the values of the gradients are too small that they, eventually, become insignificant to the model, preventing the model from further learning. On the other side, the exploding gradient problem causes the model's weights to become big values, making the learning process unstable and inaccurate. Chung et al. (2014) proposed using Gated Recurrent Units (GRUs) to address these gradient problems. This can be accomplished due to the gating mechanism within every GRU cell. These gates allow the model to learn which data in a sequence is important to keep and which data should be disposed of or ignored.

Our GRU-Spatial model uses GRUs with the input being only the spatial part of the data (i.e., only the location). Hence the temporal side of the data remains implicit to the model in the order of the input sequences. We also tested various spatiotemporal GRU architectures that used cyclical time embedding as input, including early and late fusion architectures. Still, all of them performed similarly to the GRU-Spatial model. We decided to keep only the GRU-Spatial approach in the experiments because it was smaller and faster to train than the other GRU variations, yet it achieved similar performance scores.

**Figure 1:**
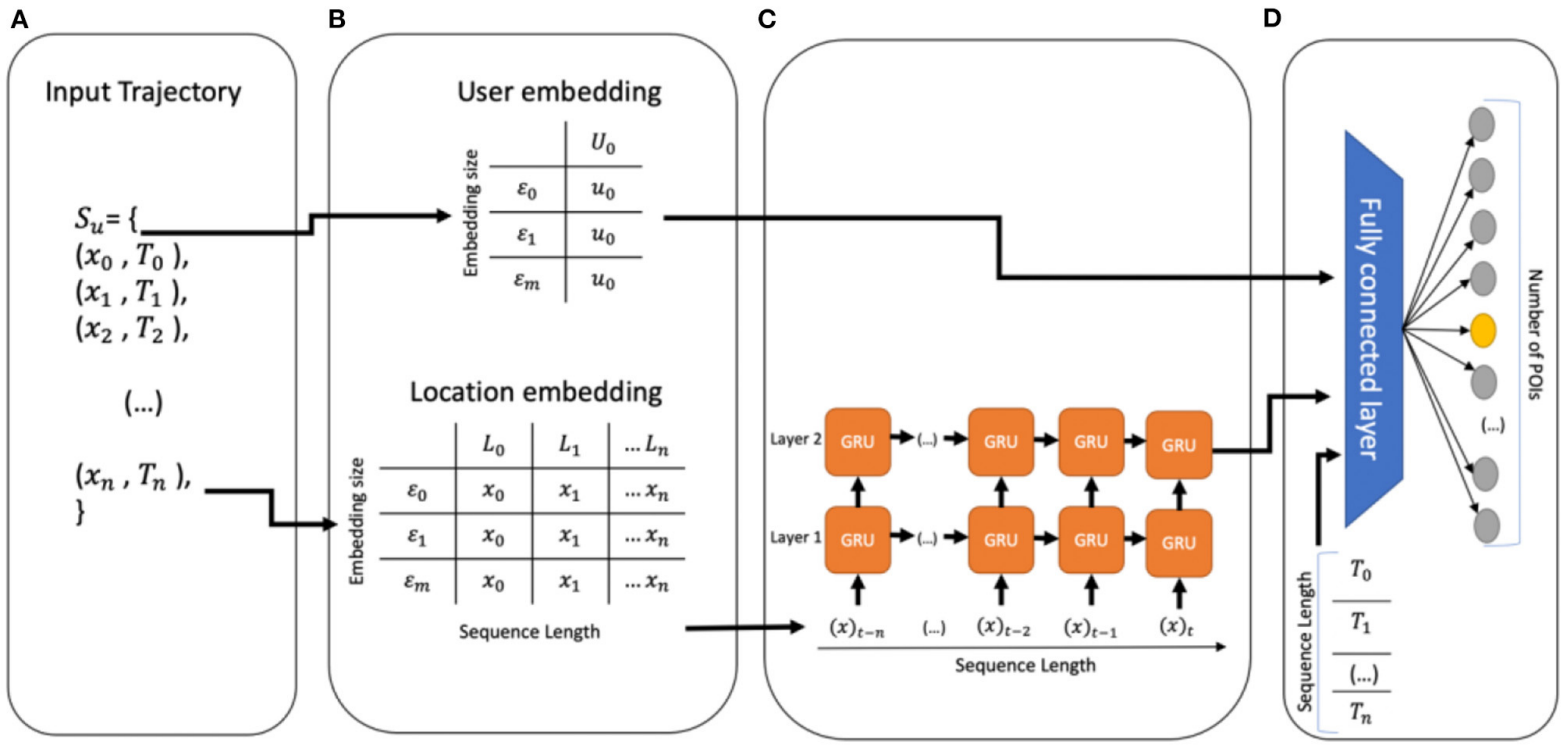
**(A)** The input of the model, only spatial data is considered, **(B)** embedding phase, **(C)** multi-layer GRU, and **(D)** fully connected layer and output.

Figure 1 depicts the GRU-Spatial architecture. It is a 2-layered GRU architecture with a many-to-many input-to-output mapping. The architecture includes:

1. **Input data:** Only the spatial data of each user is considered, as shown in Figure 1A.
2. **Embeddings:** The user and the location features are represented in a low-dimensional representation of themselves, as shown in Figure 1B. The embedding dimension for this model is of size 100.
3. **GRU sequence feeding:** The two-layered GRU uses embedded location sequences as input. The output of the last GRU layer is then passed to the fully connected layer, as shown in Figure 1C.
4. **Fully connected layer:** This layer receives the user embeddings and the output of the GRU to produce a vector of estimated probabilities with the size of the number of POIs for each given input sequence. The highest estimated probability is then considered as the predicted next location (Figure 1D).

### DeepMove Architecture

Most of the collected spatiotemporal data obtained from LBSNs tends to be sparse and incomplete. This is mainly because users must voluntarily check-in with their smartphones or devices, which most users, naturally, don't often do.

**Figure 2:**
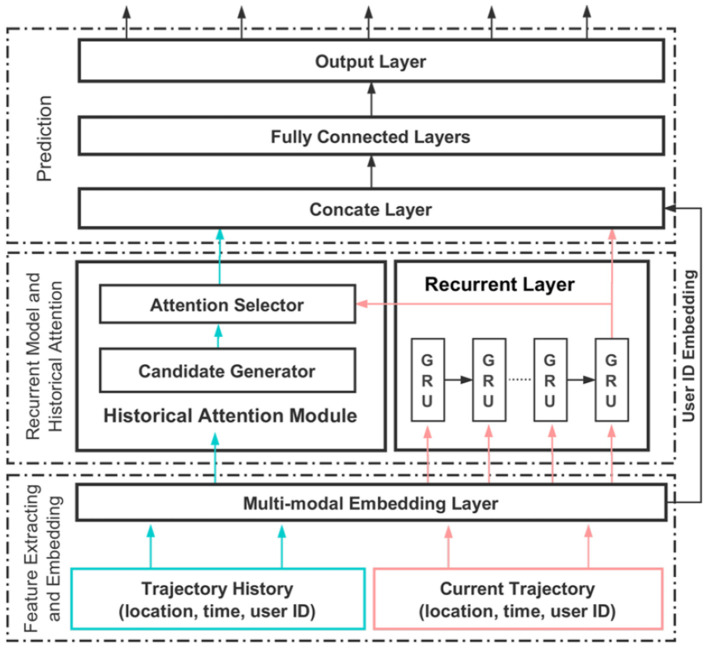
DeepMove's main architecture (Feng et al., 2018).

As shown in Figure 2, DeepMove contains three layers: (1) feature extracting and embedding; (2) recurrent model and historical attention; and (3) a prediction layer. In the first layer, a multi-modal RNN is used to capture the relationship between the transitions in the data. By doing this, Feng et al. (2018) expect to convert the sparse features (e.g., user, location, and time) into dense representations, which are more suitable for computation and have the property of expressing better meaning of the data. These representations are then fed into the RNN. By doing this, DeepMove aims to distinguish every user and learn their personal preferences while training a single model for all users

In the feature extracting and neural network phase, all trajectories are first embedded by the multi-modal embedding module. Then, these trajectories are partitioned into two parts: the current trajectory and the historical trajectory, which are processed separately in the second layer.

The recurrent layer processes the current trajectory to model the sequential information, while the historical trajectory is passed to the historical attention module to extract regular mobility patterns. The recurrent layer takes the spatiotemporal sequence embedded by the multi-modal embedding layer as input and produces an output for each hidden state. These hidden states are then called “*the current status of the mobility*”.

The historical attention module aims to capture the multi-level periodical nature of human mobility. This task is achieved by selecting the most related historical trajectories under *the current mobility status*. This historical attention module firstly extracts spatiotemporal features from the historical trajectories, and then these features are selected by *the current mobility status* to generate the most relevant context.

The historical attention module is formed by two components:

1. **An attention candidate generator** that generates candidate regularities captured from the past mobility data. For this, Feng et al. (2018) propose two approaches:

**An embedding encode module**, which directly embeds the historical records into independent latent vectors as candidate vectors.

**A sequential encode module**, which consists of a recurrent neural network. This approach takes the historical records as input and keeps the intermediate outputs of every state as the candidate vectors.

2. **An attention selector** to match the candidate vectors with the query vector by computing the similarity between the query vector (i.e., *the current mobility status*) and the candidate vector to generate a context vector.

Finally, the prediction module (layer 3) consists of a concatenation layer, several fully connected layers, and an output layer with a softmax activation.

Feng et al. (2018) suggested that the **sequential encode attention module** works better than the **embedding encode attention module** in most cases, especially in mobile data, while the latter is more computationally efficient. One reason for the better performance of the **sequential encode attention** module is its ability to capture sequential information along lengthy trajectories, while the embedding encoder cannot do this.

### Flashback Architecture

Flashback is another state-of-the-art RNN-based architecture designed to model sparse user mobility traces by doing “flashbacks” on the RNN's hidden states. In this context, Flashback can be interpreted as using spatiotemporal contexts to search historical hidden states with similar context as the current one to increase the model's predictive power.

To leverage the current spatiotemporal context for searching past hidden states, instead of feeding the RNN model with only the recurrent state (e.g., *h*_*i*_) to predict the next location (*p*_*i*+1_), Flashback computes the weighted average of the hidden states *h*_*j*_, where *j* < *i*, with a weight *W*(Δ*T*_*i,j*_, Δ*D*_*i,j*_) as an aggregated hidden state. The weight *W*(Δ*T*_*i,j*_, Δ*D*_*i,j*_) is designed to measure the predictive power of the hidden state *h*_*j*_ according to its spatiotemporal contexts. The weight can be understood from two different perspectives: a spatial perspective and a temporal perspective.

▪ **Temporal perspective:** The goal of the temporal perspective is to incorporate into the aggregated weight the periodicity of user behavior which describes how users tend to return to the locations they have visited before. For doing this, the havercosine function with bounded outputs [0,1] is used (Equation 3):

(3) $$\begin{array}{r} w_{period}\left({\Delta T}_{i,j}\right)\;=\;hvc\left(2\pi {\Delta T}_{i,j}\right) \end{array}$$

To affect the aggregated weight with the temporal perspective, a decay weight $e^{-\alpha {\Delta T}_{i,j}}$ is defined, where the returned probability exponentially decreases when Δ*T*_*i,j*_ is increased, and hence, the older the check-in is, the less impact this will have on the final prediction. As a result, the temporal perspective can be modeled by (Equation 4):


(4) $$\begin{array}{r} w_{T}\left({\Delta T}_{i,j}\right)\;=w_{period}{\Delta T}_{i,j}e^{-\alpha {\Delta T}_{i,j}}\;=\;hvc\left(2\pi {\Delta T}_{i,j}e^{-\alpha {\Delta T}_{i,j}}\right) \end{array}$$

Where α is called the temporal decay rate and controls how fast the weights decrease over time Δ*T*_*i,j*_. A value of α ≈ 0.1 is recommended. The havercosine function is defined as (Equation 5):


(5) $$\begin{array}{r} hvc\left(x\right)\;=\;\frac{1+\text{cos}\left(x\right)}{2} \end{array}$$

▪ **Spatial perspective:** Similar to the temporal perspective, but in this case, the closer a past check-in is to the current location, the more helpful it is for next-location prediction. For this, an exponentially decaying weight $e^{-\beta {\Delta D}_{i,j}}$ is defined, where a value of β ≈ 100 is recommended, producing (Equation 6):

(6) $$\begin{array}{r} w_{S}\left({\Delta D}_{i,j}\right)\;=e^{-\beta {\Delta D}_{i,j}} \end{array}$$

The combination of both spatial and temporal perspective produce the desired aggregated weight (Equation 7):


(7) $$\begin{array}{r} \begin{array}{c} W\left({\Delta T}_{i,j},{\Delta D}_{i,j}\right)\;=w_{T}{\left({\Delta T}_{i,j}\right)}^{*}w_{S}\left({\Delta D}_{i,j}\right)\\ \;=\;hvc{\left(2\pi {\Delta T}_{i,j}\right)}^{*}e^{-\alpha {\Delta T}_{i,j}}\;*e^{-\beta {\Delta D}_{i,j}} \end{array} \end{array}$$

As noted by Yang et al. (2020), the spatial distance is a determinant factor defining context similarity for the next-location prediction problem. Hence, with a large value of β the context similarity decreases when the spatial distances increase. On the other hand, the prediction performance slightly increases when decreasing the temporal decay alpha. A slow temporal decay over time allows the hidden states to contribute more to the local prediction.

### Federated Learning

FL is an approach that addresses the concerns on user privacy when training machine learning models. Centralized models need to get the spatiotemporal data from all users so that a general model can be trained. FL enables individual computing nodes (smartphones or data silos) to collaboratively learn a shared model while keeping all the training data on the local computing node. It is important to mention that in the FL process, while some devices are training a model locally, others are testing the general model. In that way, each newer version of the improved model is available right away to the users. Differential privacy methods can be applied with FL to further increase the privacy guarantees.

**Figure 3:**
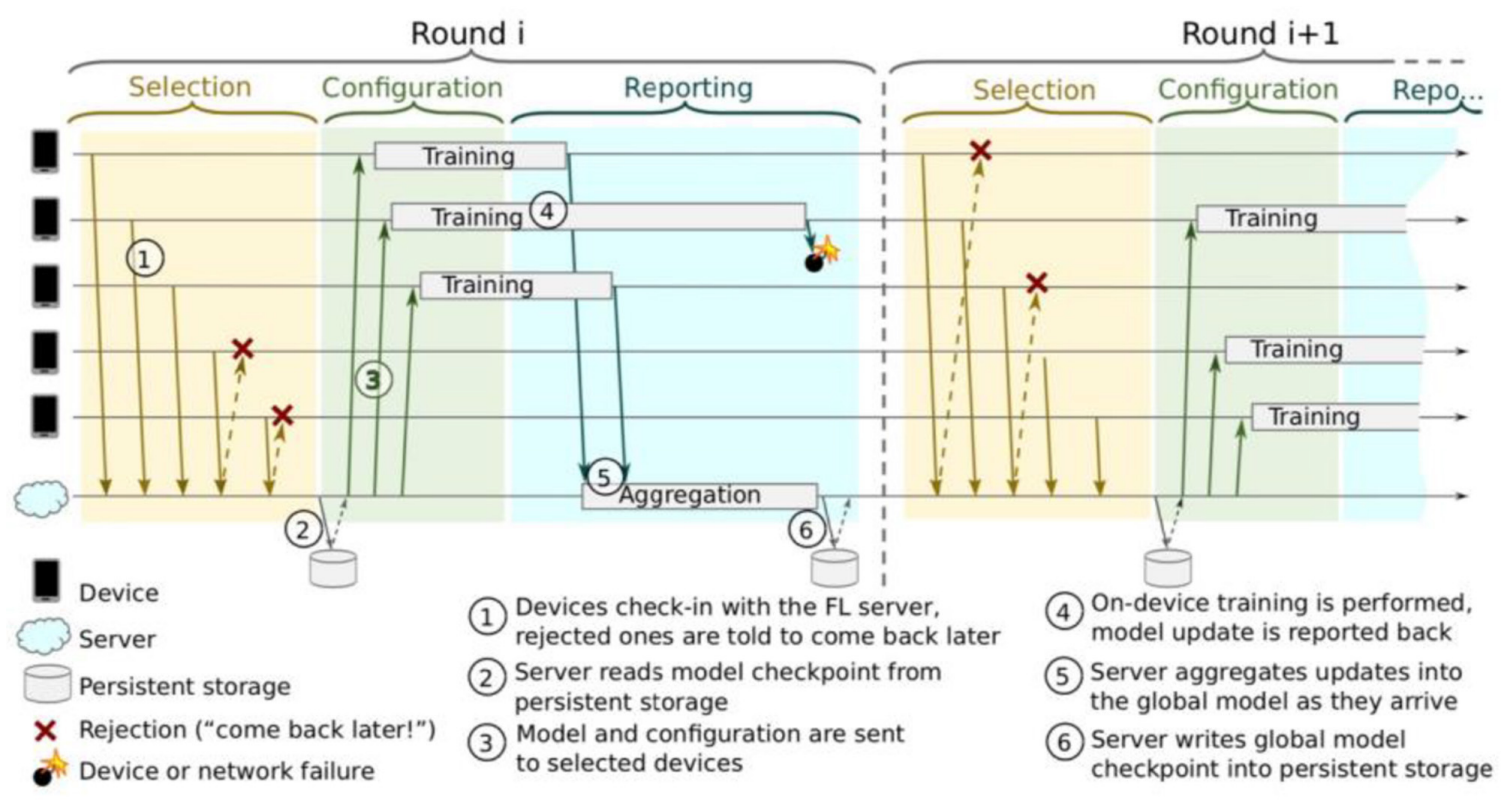
Federated Learning protocol (Bonawitz et al., 2019).

FL requires a protocol so that distributed communication can be as efficient as possible. Referring to Figure 3 by Bonawitz et al. (2019), the protocol can be divided into the following steps:

1. Each device announces to the server that they are ready to run a FL task for a given FL population. A FL task is a specific computation problem given to a FL population. The task can be training or evaluating a model locally and training with specific hyperparameters, among others. A FL population is identified by a unique ID.
2. The server selects a subset of the available devices. The selected devices stay connected to the server for the duration of the round.
3. The server specifies the task to run and provides them with a FL plan containing instructions on how to execute the task. The server then sends each participant the current global model.
4. Each participating device performs local computations based on the global model and its local dataset.
5. Each participating device sends its updated model back to the server.
6. The server incorporates the received updates into the global state, and the process repeats.

We implemented all models in this work in pyTorch, in combination with the pyTorch-friendly FL platform Flower (2021). Flower allows the creation of a centralized server that dictates the number of rounds the protocol should run and the aggregation methods to use among the other parameters. Flower also allows the creation and management of users that are communicating with the server, supporting both “simulated” user processes on the server and clients deployed on real (physical) devices. For this work, the devices were simulated as individual processes on the same PC, instead of having physical devices and real users.

On one side, the clients must receive the general model, which they will update with their data. Flower's client side is used to set up the general model's personalization and declare the critical methods for communicating with the server (loading data, training, and evaluating models). The essential methods of client are:

▪ **get_parameters:** Returns the local model weights as a list.
▪ **set_parameters:** Updates the local model weights with global weights received from the server.
▪ **fit:** Trains the global model using the local data, which produces new model weights.
▪ **evaluate:** Evaluates the local model, returning to the server the evaluation results.

On the other side, the server has the task of coordinating the actions of every client, aggregating the client model weights to a general model, and sending an updated general model to each device. The aggregation strategy used in this study is Federated averaging (FedAvg), which computes an average of the received model weights to produce a general model, which is then sent to the user devices to continue training for the next communication rounds.

## Results

This section presents the experimental setup, including hardware characteristics, hyperparameters used for training the centralized and FL models, the specific train/test split of the experimental datasets, and the evaluation metrics used to score the models. We then present the experimental results, first those achieved by the centralized models and then those achieved by the FL models.

### Experimental Setup

Before analyzing the results, it is important to consider the conditions and environment in which they were executed. This is for providing a basis for the minimal requirements for replicating the reported results:

▪ Hardware:

◦ Architecture: x86_64
◦ CPU: 6-core Intel i9-10900k @ 3.7 GHz
◦ GPU0: GeForce RTX 3080 (10,018 MiB)
◦ GPU1: RTX A5000 (24,256 MiB)

▪ Hyperparameters:

◦ Epochs: 100—centralized/10—federated
◦ Server Rounds: 0—centralized/10—federated (additional experiments with 100 and 200 rounds)
◦ Learning rate: 0.001
◦ Minimum no. check-ins per user: 100
◦ Batch size: 128
◦ Sequence length: 20
◦ Embedding size: 10

▪ Train/test dataset split:

◦ Centralized training data: first 80% of each user's data
◦ Centralized test data: last 20% of each user's data
◦ Federated training data: The centralized train data was randomly split among *n* simulated devices. The data from one user can belong to only one device. We experimented with *n* = *2* and *n* = *4* federated clients, simulating a cross-silo FL scenario. We could not perform experiments with more than 4 (simulated) clients due to memory limitations: despite having 35 GB of shared GPU RAM, the training process was causing GPU memory overflow. This was mainly due to the FL platform used for the experiments.
◦ Federated test data: The centralized test data was split among the *n* simulated devices, following the user split from the federated training data.

▪ Evaluation metrics:

◦ **Acc@K** (Equation 8)—computed as an average of how many times the correct location was within the top-k predicted places (sorted by the model's output weights). For example, for an Acc@5 metric, the target (or actual output) is compared against a vector of top-5 most probable locations output by the model. If the target is an element of the top-5 vector, the prediction is correct (or true positive). Finally, we divide the number of true positives with the total number of predictions:

(8) $$\begin{array}{r} Acc@K=\frac{True\text{\_}Positives@K}{Total\;number\;of\;predictions} \end{array}$$

◦ **Mean Average Precision (MAP)—**defined as (Equation 9):

(9) $$\begin{array}{r} MAP=\;mean\left(\frac{True\text{\_}Positives}{True\text{\_}Positives\;+\;False\text{\_}Positives}\right) \end{array}$$

◦ **Loss –** As a loss function for training and evaluating the models, we used cross-entropy loss (Equation 10):

(10) $$\begin{array}{r} L\;=\;{\left\{l_{1},\ldots ,l_{N}\right\}}^{T}\;,\;l_{n}\;=\;-\log\frac{\exp\left(x_{n,y_{n}}\right)}{\sum_{1}^{C}\exp\left(x_{n,c}\right)} \end{array}$$

where *C* is the number of classes (unique places in our experiments), *x* is the input, *y* is the target, and *N* is equal to the minibatch dimension (128 in our experiments).

### Experimental Results

#### Centralized Model Results

Table 2 shows the results of the centralized models. It can be seen that the models Flashback and GRU-Spatial performed similarly on the Foursquare dataset. On the Gowalla dataset, Flashback performed better than the other models. The DeepMove model achieved inferior results than the other two models, both on the Foursquare and Gowalla datasets. One reason for this may be the experimental setup, i.e., our experimental setup (data pre-processing and train/test split) was based on the Flashback study. This may cause an experimental bias toward the Flashback model. To verify that our DeepMove implementation was correct, we evaluated the DeepMove architecture using the same pre-processing and data split from the DeepMove study. The experimental results in these additional experiments were similar to those reported in the DeepMove study, thus confirming that our DeepMove implementation was correct. Since DeepMove performed poorly in the centralized experiments, we continued the FL experiments only with the federated implementations of Flashback and GRU-spatial.

**Table 2:** Centralized results.

**Centralized models**	**Foursquare**				**Gowalla**			
	**acc@1**	**acc@5**	**acc@10**	**MAP**	**acc@1**	**acc@5**	**acc@10**	**MAP**
GRU-spatial	23.5%	57.9%	69.4%	0.388	5.6%	15.0%	20.3%	0.105
Flashback	26.3%	55.4%	63.0%	0.394	11.6%	27.8%	35.0%	0.194
DeepMove	1.3%	2.9%	3.7%	0.002	1.8%	4.0%	5.2%	0.004

#### Federated Model Results

The initial FL experiments were performed with 10 rounds of federated training. In each round, each device was training the model for 10 epochs. These parameters enable a similar number of updates for the centralized models (100 training epochs) and the federated models (100–10 rounds × 10 epochs). The batch size was the same for all models (128).

**Federated Flashback** in Table 3 presents the evaluation results obtained by the federated Flashback model. It can be seen that the 1-client implementation achieved similar results are the centralized Flashback (see Flashback results in Table 2), confirming that the federated Flashback is correctly implemented. Furthermore, it can be seen that the greater number of clients, the lower the evaluation scores of the model.

**Table 3:** Federated results (10 rounds−10 epochs).

**Federated models**	**Foursquare**				**Gowalla**			
	**acc@1**	**acc@5**	**acc@10**	**MAP**	**acc@1**	**acc@5**	**acc@10**	**MAP**
1 client Flashback	22.3%	50.2%	58.4%	0.350	11.7%	28.1%	35.1%	0.195
2 clients Flashback	5.6%	14.4%	19.5%	0.103	5.0%	13.1%	17.9%	0.094
4 clients Flashback	3.4%	9.0%	12.8%	0.066	1.6%	4.4%	6.0%	0.034
1 client GRU-Spatial	25.0%	58.3%	68.9%	0.399	7.1%	17.9%	23.8%	0.127
2 clients GRU-Spatial	13.1%	30.1%	37.7%	0.213	2.3%	6.1%	8.5%	0.045
4 clients GRU-Spatial	9.1%	20.6%	26.2%	0.148	1.5%	3.8%	5.2%	0.028

**Federated GRU-Spatial in** Table 3 presents the evaluation results obtained by the federated GRU-spatial model. It can be seen that the 1-client implementation achieved similar results are the centralized GRU-Spatial (see GRU-Spatial results in Table 2), confirming that the federated GRU-Spatial is correctly implemented. Additionally, the same decrease in the evaluation results can be noticed as with the federated Flashback, i.e., the evaluation scores decrease as the number of clients increases. These results indicate that given the same number of model updates, i.e., 100 epochs for the centralized models and 10 rounds with 10 epochs for the FL models, the centralized models achieve better results. To analyze the training process in more detail, we also present the training learning curves of the models, i.e., the loss scores at each training epoch calculated on the training data.

##### Centralized GRU-Spatial

**Figure 4:**
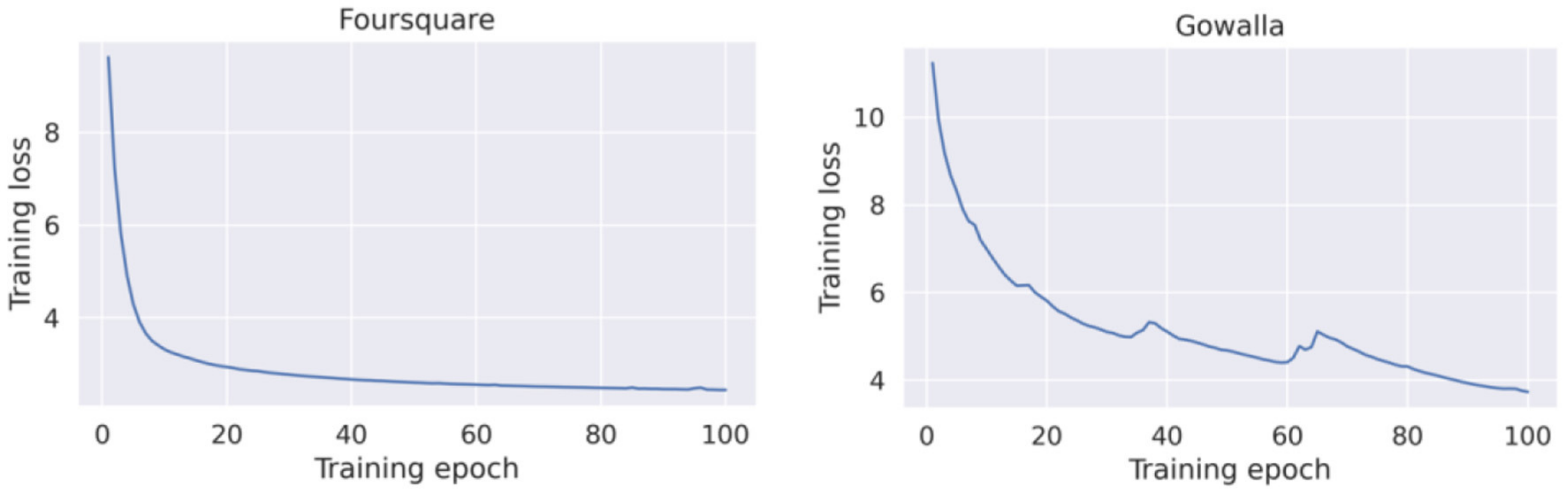
Training loss of the centralized GRU-spatial model.

Figure 4 presents the loss curves for both datasets (Foursquare and Gowalla). As a general observation, the GRU-Spatial model converged faster when training with the Foursquare data. When training on the Gowalla dataset, the model requires more training epochs to converge. One reason for this may be the sparsity of the Gowalla dataset, i.e., Gowalla is more sparse than Foursquare (see the average number of check-ins per user in Table 1). This sparsity also explains why all the models performed worse on the Gowalla dataset (results in Tables 2, 3).

##### Centralized Flashback

**Figure 5:**
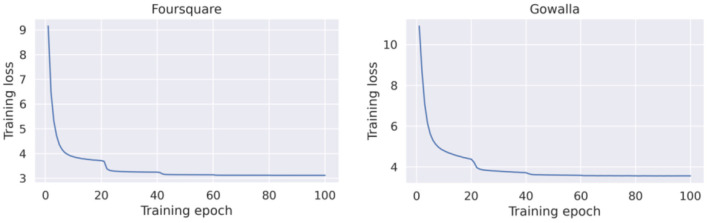
Training loss of the centralized flashback model.

Figure 5 presents the training loss curves for both datasets. Compared to the learning curves of GRU-Spatial (in Figure 4), it can be seen that the centralized Flashback converged faster for the two datasets, and the overall learning process is more stable. This is probably because the Flashback models are more complex with more training parameters that can fit the training data faster than a less complex model (e.g., the GRU-Spatial model).

##### Federated Learning GRU-Spatial

**Figure 6:**
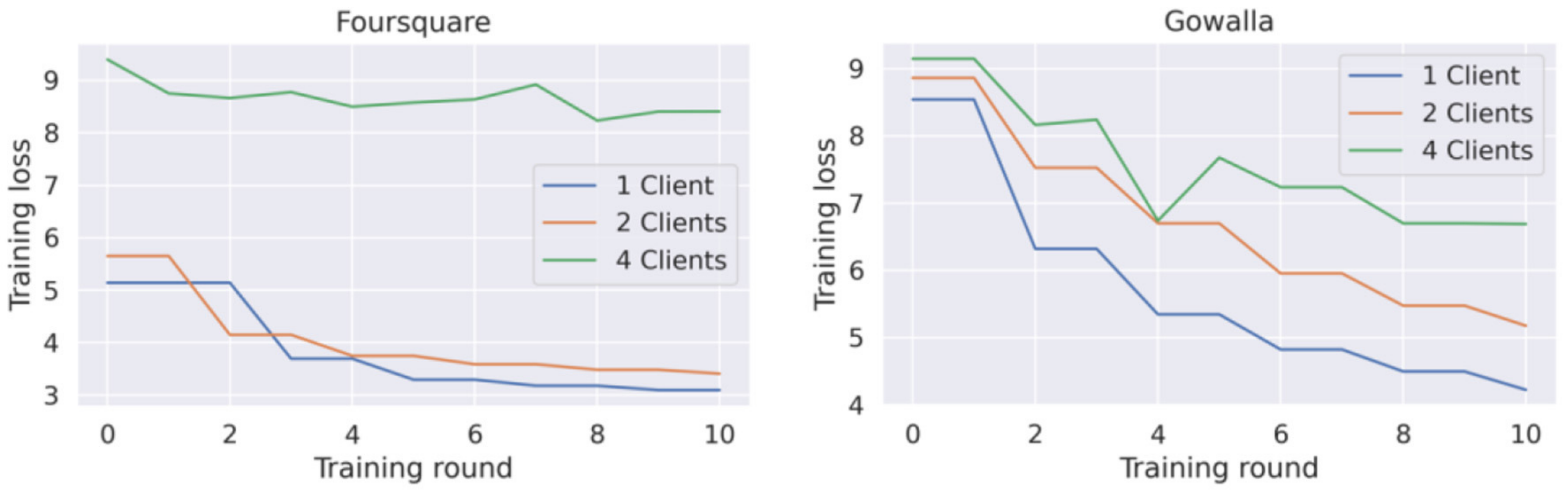
Training loss for federated GRU-spatial model with 1, 2, and 4 clients.

Figure 6 presents the training loss curve for this model's implementation with 1, 2, and 4 clients. It can be seen that the destabilization of the learning process increases each time a new client is added. This is by the accuracy reported in Table 3 for this model, which confirms that the model faces more difficulties the more clients are added to the federated training process.

##### Federated Learning Flashback

**Figure 7:**
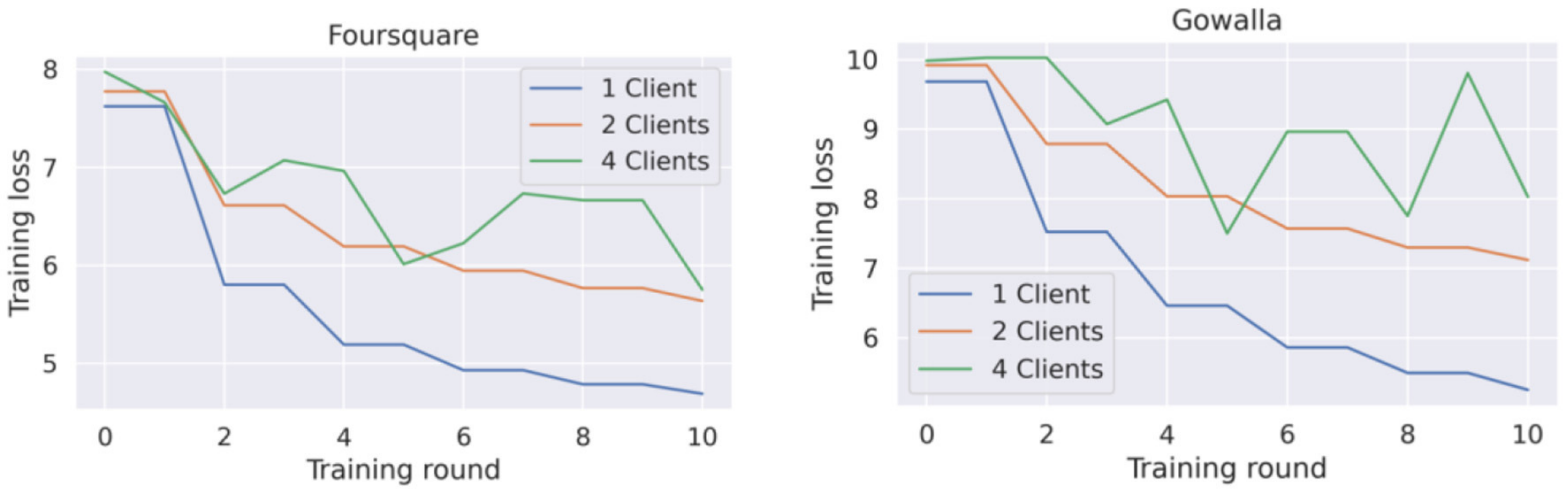
Training loss for federated flashback model with 1, 2, and 4 clients.

Figure 7 corresponds to the training loss of this federated model. Overall, the same behavior as the behavior of the GRU-Spatial model is observed, i.e., the learning curve converges slower in the scenarios with more federated clients.

**Figure 8:**
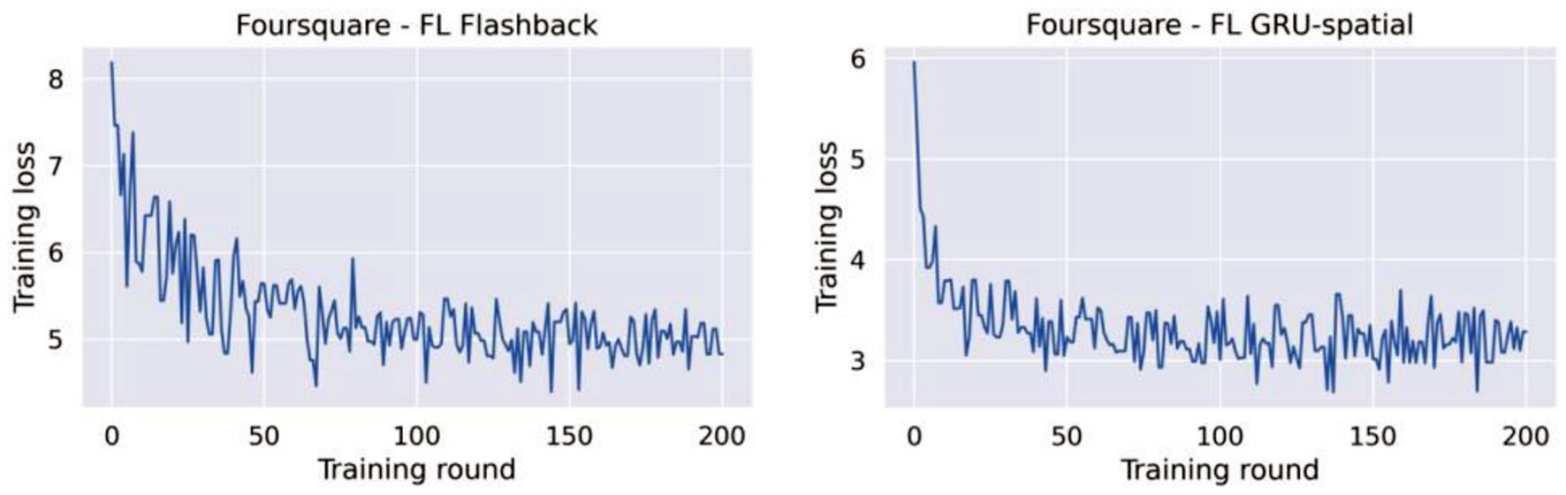
Training loss for FL-flashback and FL-GRU with 4 clients and 200 training rounds.

Another observation from the learning curves of the federated models (Figures 6, 7), is that these models did not converge, at least not to the convergence level that the centralized models did. This opened the question of whether the federated models require more training rounds to finalize their training. We performed additional experiments on the Foursquare dataset with 4 federated clients to answer this question. The models were given 200 communication rounds, instead of the 10 rounds used in the previous experiment. The number of local epochs was kept to 10 in these additional experiments. The training loss curves are presented in Figure 8. From the figures, it can be seen that on average, the training loss continuously decreases. Thus, both federated models benefit from longer training periods (more communication rounds in this case). Unfortunately, in all the cases, the training process is still quite unstable, which can be seen from the variability in the loss curves. A model with a more stable training process would also have a smoother training loss curve, similar to the learning curves of the centralized models presented in Figures 4, 5.

Additionally, Table 4 presents the test accuracies of the federated models trained for 100 and 200 rounds. It can be seen that the performance obtained after 100 and 200 rounds are very similar between them, yet, both models improved compared to the same federated models that were trained for only 10 rounds. In any case, the performance of the FL models is still lower compared to the performance of the corresponding centralized models.

**Table 4:** Accuracy for FL-GRU and FL-flashback with foursquare (10 epochs−100 and 200 rounds).

**Model**	**FL- GRU-Spatial**				**FL-Flashback**			
	**acc@1**	**acc@5**	**acc@10**	**MAP**	**acc@1**	**acc@5**	**acc@10**	**MAP**
4 clients (200 rounds)	11.33%	26.12%	33.01%	0.185	3.95%	10.52%	14.68%	0.075
4 clients (100 rounds)	10.99%	25.47%	32.33%	0.180	3.71%	9.94 %	13.97%	0.072

## Discussion

### Complexity Analysis

Table 5 presents the model sizes (through the number of trainable parameters for each model), the time required to perform one epoch over the training data, and the inference time, i.e., the time needed for each model to provide an output for a given input instance. Note that the FL models are not mentioned since their size is presumed to be the same as their centralized counterparts. The table shows that the DeepMove model is the largest, and the GRU-spatial is the smallest. This is reflected by the number of trainable parameters of each model and the training time per epoch. The size of a model is an essential factor, especially for models running on smartphones. The positive effect of having a high count of trainable parameters is that the model will learn more complex regularities. Still, on the other hand, such a model is more prone to overfitting than a less complex model.

**Table 5:** Model size, training time, and inference time for the three centralized models.

**Models**	**Trainable**	**Time**	**Inference**
	**parameters**	**per epoch**	**time**
DeepMove	109,198,710	47,371 s	0.039 s
Flashback	3,395,154	313 s	0.067 s
GRU-spatial	789,630	93 s	0.051 s

Additionally, in practice, more trainable parameters also require more computational resources, which does have an essential impact in federated applications where the trainable models must be as small as possible since it is expected that each device runs the model without affecting the end-users regular phone experience. In federated scenarios where models are retrained daily—which is different from centralized scenarios where models are trained only once in most cases—the training time is a parameter that must be considered seriously. Once trained, all models have a similar inference time.

Given that the GRU-Spatial model is 5-times smaller than the Flashback model, but performed similarly to the Flashback model on the Foursquare dataset, both in centralized and in federated scenarios, and had a slight performance decrease on the Gowalla dataset compared to the Flashback model, an important decision for real-life implementation of these models could be whether to sacrifice a bit of performance to gain computation resources, i.e., to decrease the computational complexity and free-up some device memory.

### Data Sparsity

An overall observation for both FL and centralized approaches is that the models perform better with the Foursquare dataset. This can be easily explained, given that Foursquare has almost three times as many check-ins as Gowalla (see Table 1). Although the Gowalla dataset has significantly more users and POIs, the pre-processing of the dataset (more specifically, the data reduction process) removed all users with fewer than 100 check-ins. In this way, Foursquare ultimately represents a richer dataset than Gowalla, which naturally leads to better model performance.

### Hardware and Software for Federated Learning

The FL platform used in this study, Flower, in combination with pyTorch, provides many useful tools for the advancement of deep learning and FL. Nevertheless, we faced several hardware and software challenges during the development of the FL pipelines. Despite having access to a powerful PC and two decent GPUs, we could not run experiments with more than 4 federated clients. The limitations came from the size of the dataset and the models' size. A possible solution to these problems may be dynamic loading of the training-batch data in the memory and multiprocessing solutions that enable clients to run in parallel and allocate GPU memory when needed. In this way, each simulated client (or process) can be idle or active according to the machine's capacity and the number of clients participating in the federated protocol. Flower does not directly offer the tools for multiprocessing. It treats clients as independent processes, which is suitable for prototyping FL pipelines but not optimal for emulating several clients on one machine.

### Federated Learning and IID vs. Non-IID Data

While Google's usage of FL on their Gboard application is reported to reach acceptable accuracy levels, the results presented in this work suggest a different performance for human mobility prediction tasks. The training process of the federated models was less stable, i.e., had higher variability in the loss curves, which led to a slower convergence and thus a poorer performance compared to the corresponding centralized models. The performance of the models was also highly influenced by the number of federated clients. While using validation datasets and early-stopping mechanisms may partially mitigate the problem (Goodfellow et al., 2016), other FL studies have also reported that federated approaches tend to underperform. One assumed reason for the underperforming FL models is the non-Independent and Identically Distributed (non-IID) data (Zhao et al., 2018). Having IID data on every client would mean that each client data is statistically identical to a uniformly drawn sample from the entire dataset (the union of all users). Thus, if each client's dataset was IID, the federated model could be trained as a centralized model. Unfortunately, this represents a problem for mobility datasets since assuming that the local data (i.e., individual user data) is IID is unrealistic because different people visit different POIs at different times.

To experimentally check the impact of IID vs. non-IID data, we performed additional experiments in IID and non-IID scenarios. In the IID scenario, we took the first 80% of data of the top-10 Foursquare users (ranked by the number of check-ins), and we tested the models on the last 20% of data from the same users. In the non-IID scenario, we took the overall training data (first 80% of data of all Foursquare users), and we tested on the last 20% of data of the top-10 Foursquare users. Thus, the models were evaluated using the same test data, but they were trained using different training sets. The mismatch of the users in the non-IID scenario causes the distribution of the training data and the test data to be different. Thus, the data is not identically distributed in this scenario. We trained centralized and federated GRU-spatial models. The results of these experiments are shown in Table 6.

**Table 6:** IID vs. non-IID experimental results.

**Type**	**Train data**	**Test data**	**Model**	**Acc@5**
IID	First 80% of data of the top-10 Foursquare users	Last 20% of data of the top 10-Foursquare users	Cent. GRU-Spatial FL GRU-Spatial	33% 36%
Non-IID	First 80% of all Foursquare users (including the top 10)	Last 20% of data of the top 10-Foursquare users	Cent. GRU-Spatial FL GRU-Spatial	10% 1%

The evaluation scores (Acc@5) clearly show that both the centralized and federated models outperformed the corresponding non-IID models in the IID scenario. Furthermore, both the centralized and the federated model performed similarly in the IID scenario, whereas in the non-IID scenario, the federated model performed worse than the centralized model.

## Conclusion

This study reproduced the experimental results of existing state-of-the-art end-to-end DL models for next-place prediction and compared them with novel federated implementations using two large public datasets. The main conclusions of the study can be grouped into several parts.

### Architectures

In general, the DL architectures based on RNN networks are suitable for modeling human mobility. However, it is not always the case that more complex architectures lead to better results. In our experiments, the simplest architecture, GRU-spatial, performed similarly to the more complex architecture, Flashback. Furthermore, the most complex architecture DeepMove, performed poorly compared to the other two architectures, even though it has 35x more parameters than Flashback and 100x more parameters than GRU-Spatial.

### Data Sparsity

Data sparsity is a serious problem hindering the performance of the mobility models. The experimental results clearly showed that, in general, all architectures (e.g., GRU-Spatial and Flashback), in all scenarios (federated and centralized), performed better on the experimental dataset that is less sparse (Foursquare). It is also worth considering whether this will be a serious problem in the near future with more and more people using continuous sensing daily (e.g., Google Maps).

### Centralized vs. Federated Learning

The experimental results indicate that going from centralized to federated implementations is not as simple as just changing the processing pipeline. Compared to the centralized models, the federated models had higher variability in the loss curves, which led to a slower convergence and thus a poorer performance. The performance of the models was also highly influenced by the number of federated clients and the sparsity of the evaluation dataset. These findings were consistent across the two experimental datasets.

### IID vs. Non-IID Data

Our additional experiments on IID vs. non-IID scenarios showed that both the centralized and federated models outperformed the corresponding non-IID models in the IID scenario. Furthermore, both the centralized and the federated models performed similarly in the IID scenario. In contrast, in the non-IID scenario, the federated non-IID model performed worse than the centralized non-IID model. This finding demonstrates that non-IID data is challenging both for the centralized and for the federated model. the impact of this challenge is more noticeable for the federated models than for the centralized models.

### Future Work

Future research directions include dealing with non-IID data, federated optimization algorithms, federated DL architectures, federated model selection, federated hyperparameter optimization, federated debugging, and software development platforms suitable for FL with large datasets and large models.

## Data Availability Statement

Publicly available datasets were analyzed in this study. This data can be found here: https://snap.stanford.edu/data/loc-gowalla.html and https://sites.google.com/site/yangdingqi/home/foursquare-dataset.

## Author Contributions

CE: investigation, methodology, validation, visualization, software, and writing—review and editing. MG: conceptualization, formal analysis, investigation, methodology, and writing—review and editing. ML: conceptualization, methodology, writing—review and editing, project administration, and funding acquisition. All authors contributed to the article and approved the submitted version.

## Funding

This study was funded by the Swiss National Science Foundation, project 200021_182109 (BASE: Behavioral Analytics for Smart Environments).

## Conflict of Interest

The authors declare that the research was conducted in the absence of any commercial or financial relationships that could be construed as a potential conflict of interest.

## Publisher's Note

All claims expressed in this article are solely those of the authors and do not necessarily represent those of their affiliated organizations, or those of the publisher, the editors and the reviewers. Any product that may be evaluated in this article, or claim that may be made by its manufacturer, is not guaranteed or endorsed by the publisher.
